# The genetic basis for variation in resistance to infection in the *Drosophila melanogaster* genetic reference panel

**DOI:** 10.1371/journal.ppat.1006260

**Published:** 2017-03-03

**Authors:** Jonathan B. Wang, Hsiao-Ling Lu, Raymond J. St. Leger

**Affiliations:** Department of Entomology, University of Maryland College Park, College Park, Maryland, United States of America; University of Melbourne, AUSTRALIA

## Abstract

Individuals vary extensively in the way they respond to disease but the genetic basis of this variation is not fully understood. We found substantial individual variation in resistance and tolerance to the fungal pathogen *Metarhizium anisopliae* Ma549 using the *Drosophila melanogaster* Genetic Reference Panel (DGRP). In addition, we found that host defense to Ma549 was correlated with defense to the bacterium *Pseudomonas aeruginosa* Pa14, and several previously published DGRP phenotypes including oxidative stress sensitivity, starvation stress resistance, hemolymph glucose levels, and sleep indices. We identified polymorphisms associated with differences between lines in both their mean survival times and microenvironmental plasticity, suggesting that lines differ in their ability to adapt to variable pathogen exposures. The majority of polymorphisms increasing resistance to Ma549 were sex biased, located in non-coding regions, had moderately large effect and were rare, suggesting that there is a general cost to defense. Nevertheless, host defense was not negatively correlated with overall longevity and fecundity. In contrast to Ma549, minor alleles were concentrated in the most Pa14-susceptible as well as the most Pa14-resistant lines. A pathway based analysis revealed a network of Pa14 and Ma549-resistance genes that are functionally connected through processes that encompass phagocytosis and engulfment, cell mobility, intermediary metabolism, protein phosphorylation, axon guidance, response to DNA damage, and drug metabolism. Functional testing with insertional mutagenesis lines indicates that 12/13 candidate genes tested influence susceptibility to Ma549. Many candidate genes have homologs identified in studies of human disease, suggesting that genes affecting variation in susceptibility are conserved across species.

## Author summary

We have shown that there is significant genetic variation for host defenses against the fungus *M*. *anisopliae* (Ma549) in a set of 188 *Drosophila* lines derived from nature that have been completely sequenced. This manifested as differences between lines in mean survival times, how they balanced resistance and tolerance to disease, and their microenvironmental plasticity. Despite having very different modes of infection, resistance to Ma549 is positively correlated with resistance to *Pseudomonas aeruginosa*, and resistance to the two pathogens is correlated jointly with phenotypes (sleep, oxidative stress resistance) that have been measured by other researchers. We identify a host of candidate genes associated with variation in disease resistance, many of which are known to interact physically and/or genetically enabling us to place them in a biologically informative genetic network. Overall, our results suggest that natural lines differ in their ability to control and tolerate replicating fungi during infection, which is achieved through the coordinated interplay of morphological and physiological restraints, and different immune system effectors that function in subtly different ways in different lines. Generally speaking, the results presented here can provide a starting point for further research on these important traits.

## Introduction

Fungal pathogens of insects are major regulators of insect populations, and are being developed for biocontrol of insect pests [1]. Beyond insects, fungal pathogens have an enormous influence on plant and animal life, leading to species extinctions, food security issues, and ecosystem disturbances [2]. The increased prevalence of fungal infections has stimulated investigations into antifungal immune responses in humans. A defining moment was the discovery of innate-immune Toll-like receptors in antimicrobial host defense. These were originally identified in *Drosophila* as essential components for the development of resistance to infection with *Aspergillus* (and later, other opportunistic insect pathogens) [3].

Fungi, such as *Metarhizium anisopliae* cause the majority of insect disease and play a crucial role in natural ecosystems [4]; *M*. *anisopliae* is also being developed as a biocontrol agent against fruit fly pests [5]. As most *M*. *anisopliae* strains, including the one used in this study, have a broad host range, they are unlikely to be engaging in a strict coevolutionary arms race with a particular *Drosophila* population. Using *M*. *anisopliae* in infection experiments gives us the possibility to study how hosts respond to a generalist fungal pathogen and to assess if variability among host populations is present, possibly due to divergent life histories [6]. Unlike viruses and bacteria that normally infect through the oral route, *M*. *anisopliae* breaches the cuticle reaching directly into the hemocoel using a combination of mechanical pressure and an array of cuticle-degrading enzymes [7]. We previously screened 2,613 insertional mutant *Drosophila* lines for their effects on resistance to *M*. *anisopliae* ARSEF strain 549 (Ma549) [8]. Overall, 9% of the lines had altered resistance to Ma549 indicating a large mutational target for disease resistance, and approximately 13% of these where in genes encoding immune responses including coagulation, phagocytosis, encapsulation, and melanization [8]. The non-immune genes impacted a wide variety of biological functions, including behavioral traits and nutrition.

It is generally agreed that complex traits such as disease resistance are caused by interactions between multiple gene variations and environmental factors [6]. Natural selection would weed out many of the highly deleterious mutations in the insertional mutant lines that affected disease resistance. Thus, the genetic changes with the biggest impact on disease risk are likely to occur infrequently in natural populations. A complementary approach to mutagenesis is to identify loci at which alleles with subtler effects segregate in natural populations [9]. Here, we use a community resource, the *Drosophila* Genetic Reference Panel (DGRP) [10], [11], to identify mutations associated with natural variation in disease resistance. The DGRP is a panel of inbred lines with fully sequenced genomes that was created by mating full siblings of wild-caught isofemale lines for 20 generations [10]. As experimental surrogates for individual variation, DGRP lines collectively deliver much higher statistical power compared to outbred individuals, and the lack of heterozygotes means that more extreme phenotypes may be represented in the population because rare recessives of large effect are exposed [10].

Using the DGRP, we show that wild-derived populations of *Drosophila* have substantial differences in susceptibility to Ma549, a natural fungal pathogen, and that this variation correlates with resistance to a clinical isolate of *Pseudomonas aeruginosa* (Pa14). *P*. *aeruginosa* is a quintessential opportunistic pathogen that infects a broad range of hosts, including plants and insects [12], and causes the highest human case fatality rate of all Gram-negative infections [13]. We additionally found correlations between susceptibility to Ma549 or Pa14 and several previously published DGRP phenotypes [10], [14]–[16]. We used single nucleotide polymorphisms (SNPs) and indels (hereafter collectively called polymorphisms), associated with natural variation in resistance in the DGRP to identify candidate genes. In contrast to variation in resistance to viruses [17], the majority of alleles associated with variation in susceptibility to Ma549 and Pa14 were rare. We used insertional mutagenesis lines to validate a subset of candidate genes at a high rate. Combining tagged genes from Ma549 and Pa14 GWA analyses revealed a statistically enriched network of genes involved in phagocytosis and engulfment, cell mobility, intermediary metabolism, protein phosphorylation, axon guidance, response to DNA damage, and drug metabolism.

## Results

### Quantitative genetics of disease resistance to Ma549 in the DGRP

To characterize natural variation, we quantified susceptibility to *M*. *anisopliae* (Ma549) using ~71,974 flies from 188 lines of the DGRP Freeze 2, which includes documentation of insertion—deletion polymorphisms and chromosomal inversions in addition to SNPs [11]. Age-matched flies from each line were infected topically with spores of Ma549, and survival time was monitored using three replicates (~20 flies each), per sex per line. Each line was screened this way at least twice, and ~30 lines with similar survival times were screened >3 times to validate small differences. A list of the lines used, along with LT_50_ data and *Wolbachia* status, can be found in S1 Table.

ANOVAs showed highly significant genetic variation in disease resistance (P<0.0001) between lines, with a broad sense heritability of *H*^2^ = 0.23 (*H*^2^ = 0.27) for males (females) from the pooled data (Table 1). This compares with *H*^2^ values of 0.47 (0.38) males (females) for resistance to *P*. *aeruginosa* Pa14. Disease resistance by males (females) to Ma549 was significantly [P = 0.036 (0.003)] associated with only one of the 5 major chromosomal inversions (*In_3R_K*) (S2 Table), indicative of localized LD effects. The same inversion, *In_3R_K*, also impacts disease resistance by females to Pa14 (P = 0.044). *Wolbachia pipientis* is a natural intracellular symbiont of many arthropods [18], and *Wolbachia* may confer protection against the fungus *Beauveria bassiana* in one *D*. *melanogaster* line [19]. *Wolbachia* status in the DGRP lines was without significant effect on the susceptibility of either males (P = 0.7332) or females (P = 0.8070) to Ma549, but this does not preclude an impact by *Wolbachia* on a line-by-line basis, i.e., to an individual *D*. *melanogaster* line.

**Table 1:** Analysis of variance of survival time of flies treated with Ma549 and Pa14.

Trait	Analysis	Source of Variation	df	MS	*F*	P-value	Variance
Ma549 Survival	Sexes Pooled	Sex	1	477.59	39.56	<0.0001	0.30
		Line	187	102.77	6.62	<0.0001	0.10
		Sex*Line	186	15.57	12.52	<0.0001	1.24
		Error	55216	1.24			
	Females	Line	187	52.06	56.55	<0.0001	0.33
		Error	28852	0.92			0.92
	Males	Line	186	67.68	42.37	<0.0001	0.47
		Error	26364	1.6			1.60
PA14 Survival	Sexes Pooled	Sex	1	370.74	8	0.0059	2.86
		Line	80	623.17	10.98	<0.0001	0.53
		Sex*Line	80	56.78	12.46	<0.0001	4.56
		Error	16219	4.56			
	Females	Line	80	304.05	62.2	<0.0001	3.03
		Error	7984	4.89			4.89
	Males	Line	80	385.48	90.95	<0.0001	3.75
		Error	8235	4.24			4.24

**Fig 1:**
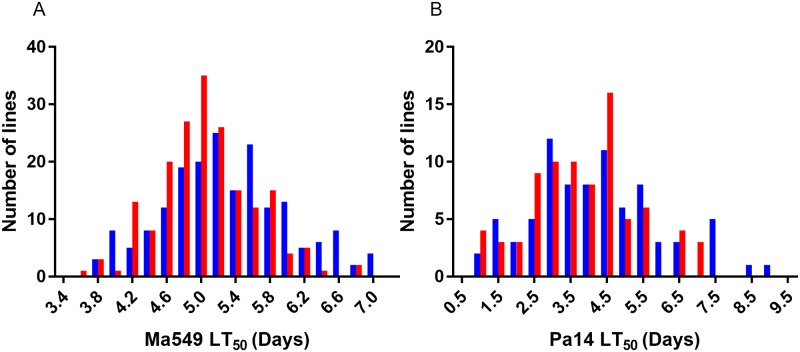
Distribution of male (blue bars) or female (red bars) lifespans among DGRP lines infected with Ma549 (A) or Pa14 (B). Lifespans were measured as the time required for half the flies to die (LT50).

The average LT_50_ for males (females) with Ma549 was 5.3 (5.1) days with a range of 3.73 (3.55) to 7.05 (6.81) days i.e. a range of 3.32 (3.26) days (Fig 1a). The mean natural lifespan in the DGRP is 55 days [20].

**Fig 2:**
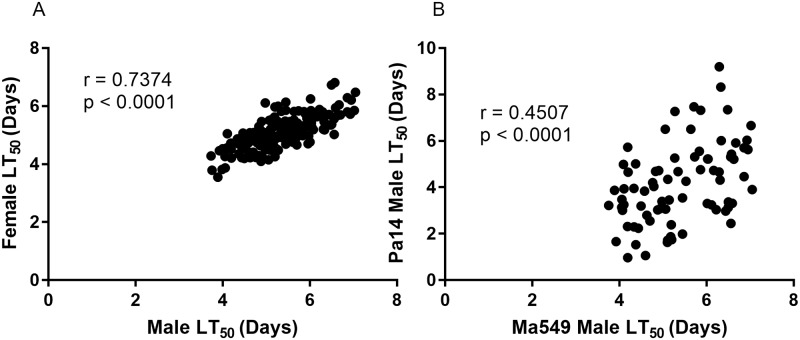
Correlation analyses of LT_50_ values among the DGRP lines between male and female flies infected with Ma549 (A), and between male flies infected with Pa14 or Ma549 (B).

**Fig 3:**
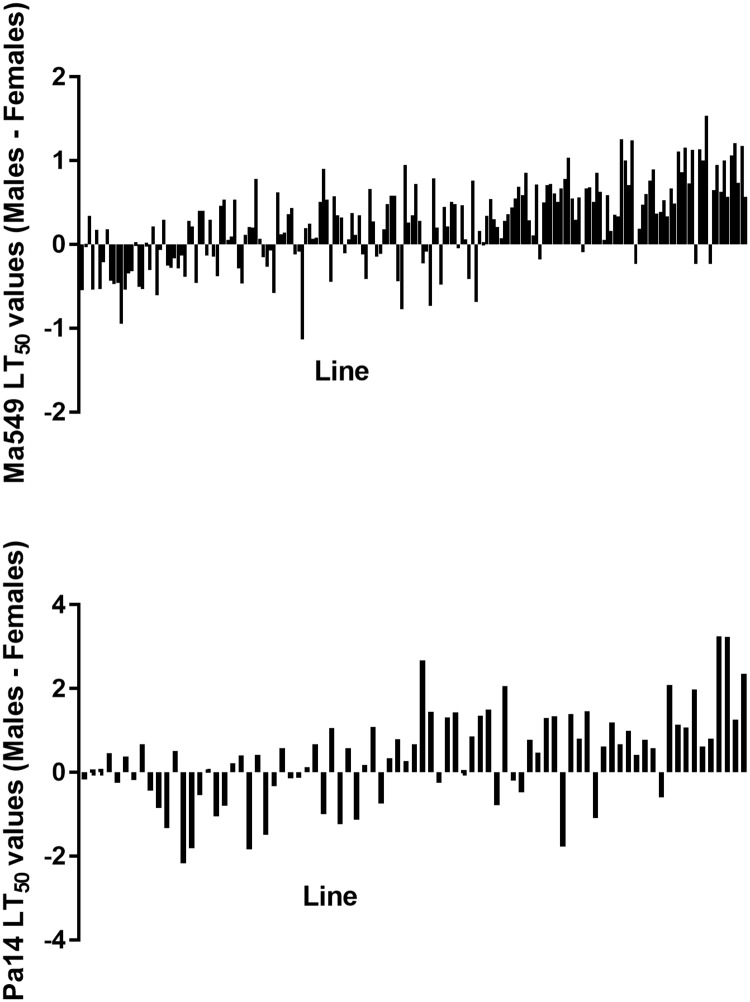
LT_50_ differences between male and female flies in DGRP lines infected by Ma549 and Pa14. LT_50_ values for females were subtracted from those of males so negative values indicate lines where female flies are more resistant than males. Lines are ranked from most to least susceptible males.

To identify sexual dimorphism, we measured disease resistance separately for males and females infected with Ma549 (Fig 2). Cross-sex genetic correlations were high (r = 0.74), indicating that many of the same variants affect disease resistance in males and females; but that some alleles will have sex-specific effects. As observed previously [8], males were typically more resistant than females (t = 7.026, P < 0.0001), however in 57 of the 188 lines (30.3%) females were more resistant (Fig 3). Of the 57 female-resistant lines, 45 (78.9%) were in the 94 most susceptible lines in the DGRP collection. Thus, females were more resistant than males in 47.9% of susceptible lines and only 12.8% of resistant lines.

### Resistance to infection by Ma549 is correlated with other phenotypes

We asked to what extent disease resistance responses to fungi and bacteria were correlated by determining LT_50_ values for a subset of 81 randomly chosen *Drosophila* lines fed food contaminated with PA14 (Fig 1b). The average LT_50_ for males (females) was 4.2 (3.8) days with a range of 0.97 (1.07) to 9.2 (6.99) days. This 8.23 (5.92) day range in variation in LT_50_ values for males (females) infected by PA14 was ~ 2.48 (1.82)-fold greater than the range for Ma549. Nevertheless, LT_50_ values for *Metarhizium* Ma549 and Pa14 were moderately correlated for both males (r = 0.45) and females (r = 0.40) (p<0.0001) consistent with *Drosophila* only partially discriminating between these pathogens (Fig 2b). Phenotypic correlations between sexes were greater than correlations between the pathogens, with r = 0.74 for Ma549 (p < 0.0001)) and r = 0.77 for PA14 (p<0.0001). The average LT_50_’s of Pa14 infected male flies (4.2 days) was significantly higher than females (3.8 days) (t = 2.96, p = 0.004). The distribution of sexual dimorphism to Pa14 was also similar to Ma549 infected lines, with females being more resistant than males to Pa14 in 30 of the 81 lines (37%) with the majority (22, 73.3%) of these being in the 40 most susceptible lines. However, the correlation of the magnitude of divergence in LT_50_’s between male and female flies infected with Ma549 or Pa14 fell short of significance (r = 0.19, P = 0.1043).

To identify trade-offs associated with disease resistance, we measured correlations between our disease resistance phenotypes and several other traits that have been measured in the DGRP and for which the data are publicly available (longevity, fecundity, courtship behavior, starvation stress resistance, nutritional stores, chill coma recovery, startle response, aggression, oxidative stress response, endoplasmic reticulum stress, sleep indices) [9], [10], [14], [16], [21]–[25]. Table 2 contains the correlation coefficient for each trait combination. Correlations between disease resistance and broad ecological measures of health such as longevity or several measures of fecundity [21], were not significantly different than zero, indicating that in a pathogen-free environment disease resistance would not be associated or traded off against general robustness or lifetime fitness. Some weak but significant associations did not pass a Holm-Bonferroni correction for multiple testing e.g., male courtship behavior and the startle response (Table 2).

**Table 2:** Correlations of LT50 values among the DGRP lines for *M*. *anisopliae* Ma549 and *P*. *aeriginosa* Pa14 (this study) with traits previously measured by other groups.

	Ma549		Pa14	
PHENOTYPES	Male	Female	Male	Female
Male flies vs. Ma549 LT50	1	**0.74**^c^*	**0.45**^c^*	**0.48**^c^*
Female flies vs Ma549 LT50	**0.74**^c^*	1	**0.31**^b^*	**0.4**^c^*
Male flies vs Pa14 LT50	**0.45**^c^*	**0.31**^b^	1	**0.77**^c^*
Female flies vs Pa14 LT50	**0.48**^c^*	**0.4**^c^*	**0.77**^c^*	1
***Overall Fitness***				
Lifespan LS Mean	n/a	0.03	n/a	0.17
Wk1 Fecundity LS Mean	n/a	-0.07	n/a	0.11
Wk3 Fecundity LS Mean	n/a	0.07	n/a	0.03
Wk5 Fecundity LS Mean	n/a	0.06	n/a	0.08
Wk7 Fecundity LS Mean	n/a	0.09	n/a	-0.06
Life Time Fecundity LS Mean	n/a	-0.01	n/a	0.11
Courtship Behavior	**0.17**^a^	n/a	0.04	n/a
***Response to Stimuli***				
Startle Response	0.1	**0.17**^a^	0.07	0.03
Startle Response (MSB)	0.14	**0.18**^a^	0	0.03
Negative Geotaxis	**0.2**^b^	**0.2**^b^	0.06	**0.26**^a^*
Aggression	0.04	n/a	0.11	n/a
***Response to Stress***				
Paraquat resistance	0.13	**0.31**^c^*	**0.36**^b^*	0.17
MSB resistance	-0.06	0.07	0.18	0.01
ER Stress Hazard Ratio	0.15	n/a	0.2	n/a
ER Stress T50	0.05	n/a	0.07	n/a
Chill Coma Recovery	0.08	-0.05	0.13	0.21
***Nutritional Status***				
Starvation Resistance	0.1	**0.16**^a^	**0.27**^a^*	**0.28**^a^*
*High Glucose Diet*				
Blood Glucose	0.07	n/a	0.02	n/a
Weight High Glucose Diet	0.05	n/a	0.04	n/a
*Low Glucose Diet*				
Blood Glucose	**0.17**^a^	n/a	**0.31**^a^*	n/a
Triglyceride Low Glucose Diet	**-0.18**^a^	n/a	-0.12	n/a
Weight Low Glucose	0.08	n/a	0.17	n/a
***Sleep indices***				
Night Sleep Duration (Min)	**-0.32**^c^*	**-0.28**^c^*	-0.01	-0.14
Day Sleep Duration (Min)	**-0.2**^a^	0.04	-0.05	-0.05
Night Bout Number	**0.25**^b^*	**0.24**^b^*	-0.04	**0.27**^a^*
Day Bout Number	0.12	**0.16**^a^	-0.03	0
Night Avg. Bout Length (Min)	**-0.2**^a^	**-0.21**^b^	-0.02	**-0.29**^a^*
Day Avg. Bout Length (Min)	**-0.19**^a^	-0.05	0	-0.14
Waking Activity	-0.12	**-0.2**^a^	0.09	0.07

^a^P < 0.05,

^b^P < 0.01,

^c^P < 0.001.

* Passes Holm-Bonferroni method

n/a = trait not studied in that sex

Negative geotaxis (a measure of innate escape response and general stress resistance) as determined by Jordan et al., [14] is positively correlated with resistance to Ma549 in both males (r = 0.2) and females (r = 0.2) (P < 0.01), but was only correlated with the resistance of female flies to Pa14 (r = 0.26, P < 0.05). Negative geotaxis has been shown to be sensitive to oxidative stress [14]. Sensitivity to oxidative stress, induced by paraquat but not menadione sodium bisulfate (MSB) [25], was positively correlated with the resistance of female flies to Ma549 (r = 0.31 P < 0.001) and male flies to Pa14 (r = 0.36, P *<* 0.001).

Resistance to starvation [10] is positively correlated with resistance to PA14 in both males (r = 0.27) and females (r = 0.28) (p< 0.05), but was only correlated with the resistance of female flies to Ma549 (r = 0.16, P < 0.05), indicating that Pa14 causes greater nutrient stress to *Drosophila* than Ma549. However, disease resistance was not associated with wet weight of the fly lines so larger flies are not necessarily more resistant. Various measurements of energy reserves by Unckless et al., [16], such as glycogen stores, total triglycerides and soluble proteins in flies showed no correlation with disease resistance, suggesting that there is no straightforward association between these traits. Unckless et al., [26] found that bacterial (*Providencia rettgeri*) loads were negatively correlated with blood glucose levels. Conversely, we found resistance to Pa14 in male flies (Unckless et al., [16] only tested males) was positively correlated (r = 0.31, P < 0.05) with glucose levels in flies fed a low glucose diet.

Resistance to Ma549 was negatively correlated with sleep duration, particularly at night in males (r = -0.32) and females (r = -0.28) (P < 0.001) and to a lesser extent, and then only in males, during the day (r = -0.2, P < 0.05). Conversely, there was a positive association between resistance and the number of sleep bouts in males (females) of 0.25 (0.24) (P < 0.01). Similarly, resistance of female flies to Pa14 was positively correlated with number of nocturnal sleep bouts (r = 0.27, P< 0.05) and negatively correlated with average bout length (r = -0.29, P< 0.05). Hence there is a trend for resistant flies to have more sleep bouts than susceptible flies, but these bouts are shorter and total sleep time is less. This may be related to findings that the phagocytic activity of *Drosophila* immune cells is circadian-regulated and peaks at night during the night rest [27]. However, our data suggests that the number of sleep bouts has more effect than sleep duration on resisting infections with Ma549.

Our measurement of longevity in 20 lines was moderately (r = 0.52, P < 0.05) correlated with that of Durham et al., [21], indicating the genetic robustness of phenotypes across lab groups and different assay conditions (we used batches of flies grown on cornmeal-molasses-yeast-agar medium with Tegosept and propionic acid, whereas Durham et al., [21] used pairs of flies kept separately and grown on sucrose-yeast agar). However, resistance to Pa14 was not significantly correlated (r = -0.21, P = 0.12, n = 58) with resistance of female flies to *Pseudomonas entomophila*, even when we expressed our data as % killed in 3 days (r = 0.22, p = 0.10, n = 58) following Sleiman et al [28]. Using this metric (% killed) for our data we lost correlations with sleep indices. As the specialized entomopathogen *P*. *entomophila* relies on novel secondary metabolites and toxins to kill insects [29], we speculate that the Sleiman et al., [28] analysis may have included measuring variation in resistance to these.

The absence of overall positive or negative correlations between resistance and most metabolic indices does not exclude trade-offs in individual fly lines, as all these parameters are complex traits and the product of pleiotropic genes. Thus, polymorphisms associated with increases (decreases) in disease resistance are not consistently associated with increases (decreases) of resistance to oxidative stress, starvation stress, nutrient levels, fecundity etc. S3 Table shows a subset of the 10 most Ma549 resistant and 10 most Ma549 susceptible DGRP lines (hereafter called the “divergent subset”), and their life cycle parameters and rankings in correlated data from other groups. RAL-38, the most resistant line to Ma549, ranked 154 out of 167 for resistance to paraquat, whereas the 3^rd^ and 5^th^ most resistant lines ranked 33rd and third, respectively. Thus, resistance to oxidative stress may be a factor in resistance of some fly lines but not others. While there is no significant correlation between MSB resistance and resistance to Ma549 in female flies, a t-test comparison of the absolute rankings of the divergent subset for MSB resistance reveals significant differences in survival time to MSB. Similarly, there are lines with increased levels of resistance to Ma549 and starvation stress, sleep duration or nutrient levels, but there are also resistant lines with moderate or low rankings for these indices. Consequently, overall correlations could be non-significant for some indices if there are pleiotropic effects of polymorphisms affecting disease resistance on other traits, but the effects are not in the same direction.

### The impact of natural host variation on fungal fitness

To further investigate the impact of natural host variation on Ma549 fitness we compared a subset of 20 divergent lines (S3 Table), for differences in impact on four key Ma549 life history traits at different steps of the infection process; within-host growth (fungal load, measured as CFU’s), host life span (LT_50_ values), latent period (the lag time between inoculation and sporulation), and sporulation capacity (the total number of spores per *Drosophila* cadaver).

**Fig 4:**
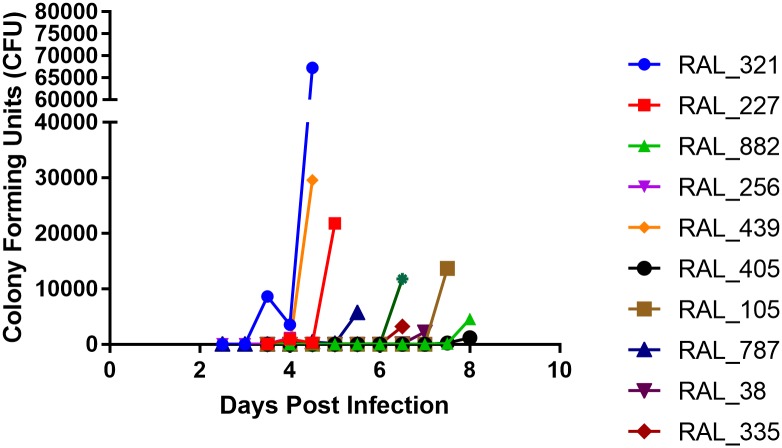
Time course of CFU production in male flies from DGRP lines. Flies were homogenized and plated at 12 h intervals post-infection until death. Ten lines are shown as representative examples. CFUs were averaged from ten individual flies per fly line per time point.

A time course of CFU counts showed that resistant flies delayed fungal growth compared to susceptible flies (Fig 4). Absolute numbers of viable fungi recovered after infection from hemolymph differed substantially between different lines and did not necessarily correlate directly with lethality (LT_50_). However, in all lines, except for the susceptible line RAL_439, fungal loads increased in the 36 hours preceding death. Consequentially, there was a strong association between LT_50_ values and the time points at which flies carried fungal loads of >10 CFUs (r = 0.61, P = 0.0086) or >100 CFUs/fly (r = 0.82, P = 0.002).

**Fig 5:**
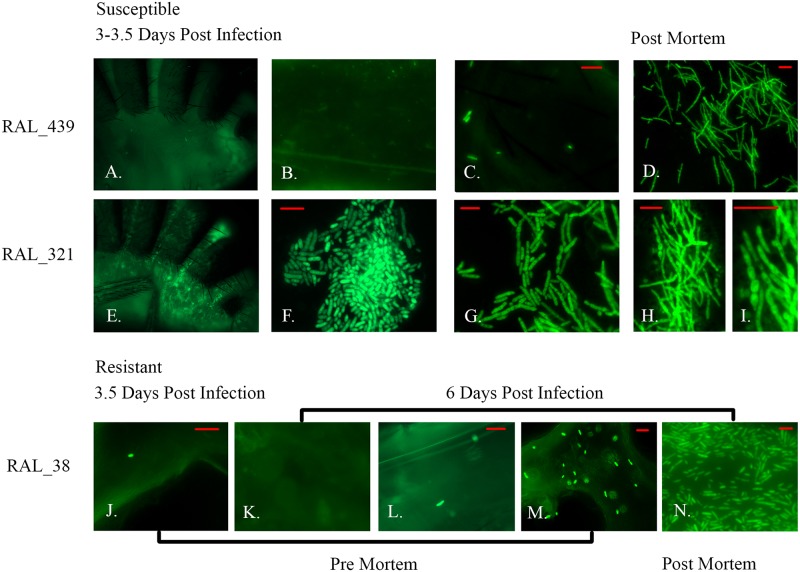
Growth of GFP-expressing Ma549 can be visualized in DGRP flies. DGRP fly lines RAL_439, RAL_321 and RAL_38 photographed at different time points post infection with Ma549-GFP. A) No visible growth of Ma549-GFP in a living RAL_439 fly’s abdomen, and there are very few pre-mortem fungal propagules in squashed RAL_439 flies (B-C). Ma549-GFP only proliferates in RAL_439 post mortem (D). In contrast, Ma549-GFP blastospores and short hyphal lengths are visible in the hemocoele from outside a still living RAL_321 fly’s abdomen (E), and in pre- and post-mortem squash preparations (F to I). Variation among individual flies in resistant line RAL_38 in number of Ma549-GFP propagules per fly 6 days’ post-infection (J-N). At death, Ma549-GFP blastospores and short hyphal lengths are found in hemocoele of all DGRP lines (D, H, I and N). The pictures are representative of the 10 flies per line per time point examined for the experiment. Bars in images represent 20 μm.

We also used a Ma549 transformant expressing GFP (Ma549-GFP) to track infections in whole insects and hemolymph in the 20 different lines (Fig 5 for exemplar images). Ma549-GFP is sufficiently bright as to be clearly visible from outside the infected insect’s abdomen, which confirmed that blastospores and hyphal bodies accumulated in the body cavity in the day preceding death. Consistent with CFU counts, the timing of colonization and the fungal load in the hemocoel are affected by the fly’s genetic background (Fig 5), indicative of micro-environmental plasticity. Fluorescence showed blastospores (yeast-like budded cells thought to be important for dissemination of the pathogen) appearing in the hemolymph ~day three in most susceptible lines, as illustrated by RAL_321 (Fig 5). In contrast, proliferation of blastospores and subsequently elongated ellipsoid cells only occurred 4 to 4.5 days post-infection in resistant fly lines, demonstrating a longer time lag between penetration and proliferation than occurred in susceptible lines. However, we also noted differences in fungal behavior in different fly lines, even where these had very similar LT50’s. Ma549 produced very few (<5) blastopores in the susceptible line RAL_439 three to 3.5 days post infection when flies were already dying. In contrast, the slightly less susceptible line RAL_321 contained abundant blastospore’s 3.5 days post infection (average 8,600 CFU counts/fly), and at time of death, these had differentiated into hyphal filaments with simple branching (Fig 5). These filaments consisted of chains of budding cells marked by constrictions rather than septa at the junctions, and thus fit the definition of pseudohyphae [30]. The proliferation of hyphal chains before fly death would result in CFUs underestimating the number of fungal cells in hemolymph. In most lines, long hyphal lengths accumulated in the body at or after death. This probably reflects different environments in line RAL_321 and the other lines in the day preceding death but the nature of the environmental signals that control the ability of Ma549 to form blastospores or pseudohyphae is unknown.

Spore production is a measure of pathogen transmission potential and therefore pathogen fitness [8]. Host genotype impacted the onset of Ma549 sporulation (latent period) which moderately correlated (r = 0.51, P < 0.01) with life span. This is readily explained by sporulation only commencing on cadavers within 60 hours’ post-mortem. However latent period was not associated with total spore production. Indeed, we found no significant difference (P = 0.26) in spore production per cadaver between the 10 most resistant (1.86 x 10^7^ ± 1.94 x 10^6^) and 10 most susceptible (1.67 x 10^7^ ± 1.48 x 10^6^) lines in the divergent subset (S3 Table), indicating that rapid kill of susceptible hosts will not be disfavored by natural selection because it is traded off against reduced time to exploit host nutrients for substantial pathogen reproduction.

### Micro-environmental plasticity in GWA lines

**Fig 6:**
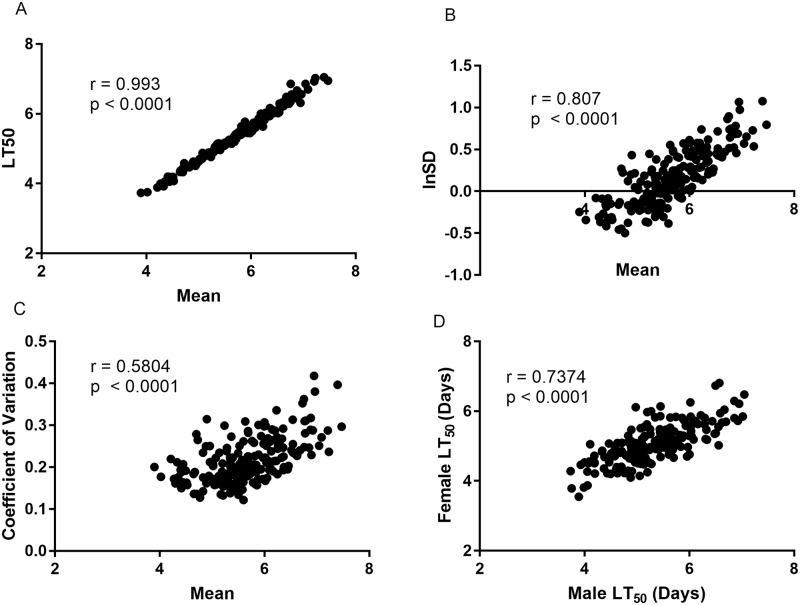
Correlation analyses in DGRP lines challenged with Ma549: (A) LT_50_ versus mean survival times; (B) ln(SD) versus mean survival times; (C) coefficient of variation (*CV*_*E*_) versus mean survival time, and (D) *CV*_*E*_ versus ln(SD).

**Fig 7:**
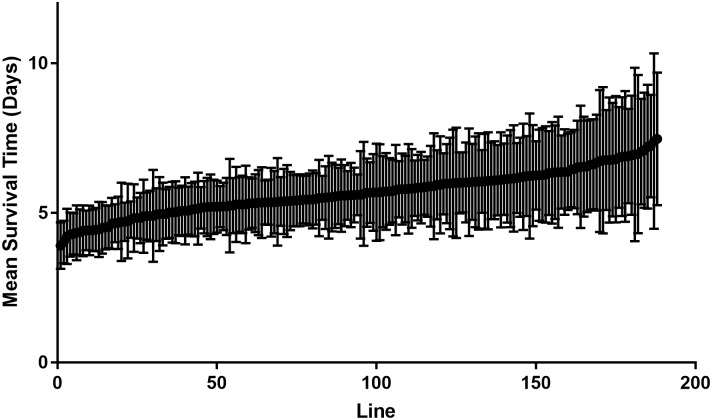
Male mean survival times of DGRP lines and their standard deviations when treated with Ma549.

To quantify micro-environmental plasticity (variation among individuals of the same genotype reared in a common environment), for mean times to death values we used the within-line standard deviation (*σ*_*E*_, and its natural log ln(*σ*_*E*_), and the within-line coefficient of environmental variation (*CV*_*E*_, ln(*CV*_*E*_)) (Figs 6 and 7). The number of segregating sites and standard deviation per fly line were not correlated (r = 0.07, p = 0.354) which suggests residual heterozygosity does not contribute to within line standard deviation. *CV*_*E*_ is often used to remove any relationship between mean and variance, but ln(*σ*_*E*_) has other advantages [31], so we used both metrics. The correlations between ln(*σ*_*E*_) and *CV*_*E*_ are high in Ma549 infected flies (r = 0.94, p <0.001) (Fig 6). Likewise, LT_50_ values and mean survival times were highly correlated (r = 0.99, p <0.0001) (Fig 6a). With either metric, disease resistance was highly variable among flies with identical genotypes (Fig 7), suggesting that some lines are relatively more canalized and others more phenotypically plastic in response to the same random environmental effects [32]. Genetic correlations show that the micro-environmental plasticity (*σ*_*E*_ or *CV*_*E*_), was most variable in lines having the highest LT50 or mean survival times (Fig 6B and 6C) suggesting that some of the variants affecting the mean also affect the micro-environmental variance. As reported for stress responses [31], the magnitude of the genetic variance affecting micro-environmental plasticity is high, with broad sense heritability’s (*H*^2^) of ln(*σ*_*E*_) of *H*^2^ = 0.5 (Ma549) and *H*^2^ = 0.52 (Pa14). Thus, the broad sense heritability at the variance level for resistance to Pa14 is of the same magnitude as that at the level of the mean and, for Ma549, the heritability of micro-environmental variance is twice as large as that of the mean.

### Genotype-phenotype associations

**Fig 8:**
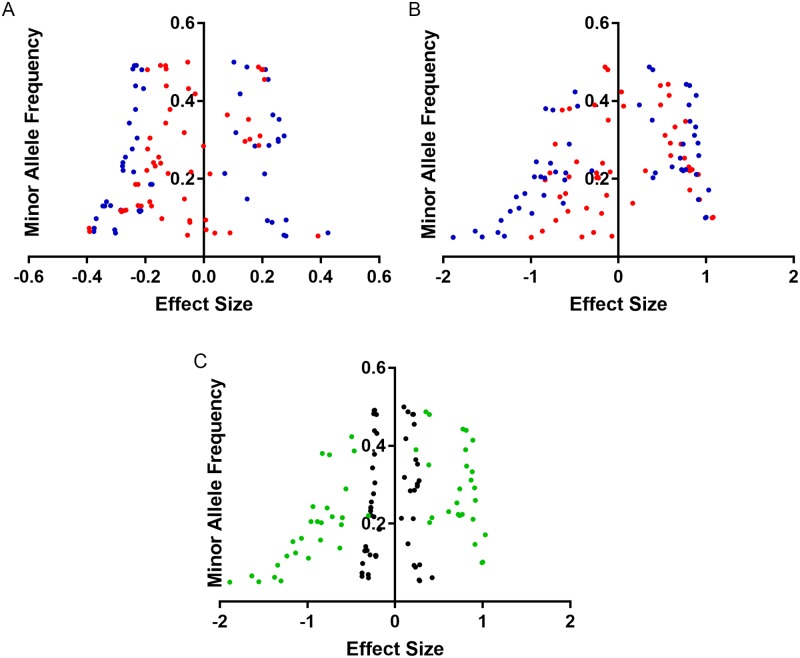
Minor allele frequency versus effect size for females (red) and males (blue) infected by Ma549 (A) or Pa14 (B), and comparing Ma549 (black) and Pa14 (green) infected male flies (C).

**Fig 9:**
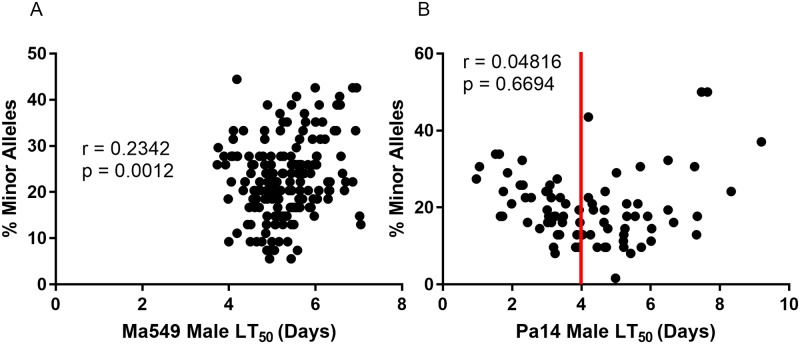
Mean LT_50_ per line plotted against number of minor alleles per line for Ma549 (A) and Pa14 (B). The red line indicates the inflection point of the parabola.

To identify genes that harbor alleles that confer altered susceptibility, Ma549 and Pa14 mean LT50s were plugged into the DGRP pipeline with a discovery *P* value <10^−5^. Most polymorphisms associated with mean time to death were at the low range of the allele frequency spectrum, with frequencies below 0.2 for 44% (41%) of Pa14 (Ma549) alleles (Fig 8). These lower frequency alleles had larger effects on LT50 values than common alleles (Fig 8), consistent with GWA studies on some other complex traits in the DGRP population [25], [33]. Negative effects (where flies homozygous for the minor low frequency allele live longer following infection than do flies homozygous for the major allele), greatly outnumbered positive effects. A corollary of this is that the most Ma549 resistant DGRP lines had a preponderance of low-frequency alleles (r = 0.23, P < 0.0012) (Fig 9A). The effect was more complicated for Pa14 [where there was no overall correlation (r = 0.05, P = 0.67)] and the distribution of minor alleles traced a parabola (Fig 9B). The heritability of Pa14-induced mortality was analyzed on 81 lines only, which means there may be a higher level of false associations [14 (>20%) of the polymorphisms have a minor allele count of 5 or less (S4 Table)]. However, if susceptible lines (LT50’s < 4 days) and resistant lines (LT50’s > 4 days) were considered separately the associations were r = -0.65 (P < 0.0001) and r = 0.45 (P 0.0034), respectively, consistent with minor alleles being concentrated in the most susceptible as well as the most resistant lines.

Pa14 polymorphism effects were much larger than those observed in Ma549 (Fig 8C), consistent with the much greater variation between lines observed in Pa14 LT_50_ values (Fig 1). Likewise, male polymorphism effects with Ma549 or Pa14 were larger than those observed in females (Fig 8a and 8b). The majority (63%) of Ma549 tagged polymorphisms had sex-specific effects, with the greater number (20) effecting survival of males as compared to 14 for females. This is consistent with the mean differences in male and female survival (Fig 1), but contrasts with alleles conferring genetic risk to oxidative stress where SNP effects were larger in females than males [25]. For Pa14, 14 mutants had female-specific effects, and 17 mutants had male-specific effects.

### Polymorphisms associated with resistance to Ma549

SNPs/indels that are significantly associated with variation in LT50’s to Ma549 and Pa14 (P < 10^−5^) are presented in S4 Table. We found 50 SNPs and 4 indels associated with Ma549’s speed of kill with a discovery P<10^−5^ (45 total associated genes). With a more stringent cut off of P<10^−6^, there were four top-candidate genes (*hig*, *Cyp4p2*, *msn*, and *Rab26*). Overall, polymorphisms significantly associated with variation in disease resistance are disproportionately found in introns and UTRs, as opposed to synonymous substitutions or positions more than 1000 bp from known genes. For example, of the 54 candidate polymorphisms, five were indels (three in introns, two within 1000 bps downstream of a gene) and 49 were unique SNPs. Of these SNPs, four were synonymous, five mapped to within 1000 bps downstream of a gene, 9 were intergenic (more than 1000 bp from known genes), three were non-synonymous, two were in a 5’UTR and the remaining 26 were intronic. Fourteen polymorphisms are located in overlapping genes that could affect either or both genes. Thus, for Ma549 resistance, 34 out of 54 (63%) total significantly associated polymorphisms are found in introns, UTRs, or as nonsynonymous SNPs, and 45 (83.33%) overall were genic. Given the percentage composition of the *Drosophila* genome (48.2% genic (including 18.3% exonic and 30% intronic) and 51.8% intergenic [34], this enrichment for putatively functional polymorphisms is significant (χ2 = 4.714, df = 1, P < 0.03). Each polymorphism that associates significantly with variation in a measured phenotype is given in S4 Table, including significance level, estimated effect size, minor allele frequency and type of polymorphism.

Of the 45 candidate genes, 34 (75.6%) have human counterparts, and 22 (48.9%) have human counterparts associated with disease (S5 Table). *hig*, *Cyp4p2*, *jhamt*, *Mctp*, *tRNA*:*CR30229*, *sickie*, *CG12344*, *CG13229*, *CG33172*, *CG17209* had multiple significant polymorphisms affecting resistance to Ma549. With the singular exception of one of the four polymorphisms in *CG13229*, the effects of these polymorphisms in each gene were in the same direction implying LD in variable genes. All four synonymous and intron polymorphisms in *hig* had a positive effect (lines homozygous for the major allele survived infection longer than flies homozygous for the minor allele), and the best-supported SNP had a nominal P-value of P = 3.18x 10^−7^. Aside this synonymous SNP, the other three SNPs were in the same intron and between 411–442 base pairs upstream of the nested gene *Cyp4p2*. None of the SNPs outside the *hig* gene localized to genes surrounding *hig* so this area of strong linkage disequilibrium only extends across the *hig* gene. *Cyp4p2* is involved in resilience to sleep deprivation and wakefulness [35]. Being involved in the functioning of cholinergic synapses, *hig* is also required for wakefulness, and deficiency mutations show severely reduced activity and longevity [36][37]. Both polymorphisms in *jhamt* (hormone secretion) had a negative effect. Two polymorphisms in *Mctp* (calcium ion binding) had negative effects; the *Drosophila* gene has no reported function but its human homolog is implicated in oxidative stress and disorders in eating [38].

Most of the genes affecting response to Ma549 have not been previously implicated in interactions with *Drosophila* pathogens. Overall, they fall into 11 ontological categories with reported roles in defense, metabolism, morphogenesis and development, stress responses, cellular communication, behavior, and gene expression. Immune genes include *sickie* required for activation of Relish, an Imd signaling component involved in antibacterial and antifungal polypeptide induction [39], and *CG5794/puffeye* (*puf*), a ubiquitin-specific protease that is a negative regulator of Imd and Toll innate immune defenses; its human homolog also plays regulatory roles in immune signaling [40].

Many of the candidate genes are pleiotropic with functions in cell adhesion and epithelial wound repair plausibly linked with infection, and some may link the immune system, nervous system and nutrition e.g., *Neuroglian* (*Nrg*) is involved in neuron cell-cell adhesion as well as melanotic encapsulation of parasites [41]. Likewise, *Lar* is involved in multiple processes involving cadherin and adhesive interactions [42]. The cadherin *Dystroglycan* (*Dg*) is associated with stress responses in *Drosophila* and humans [43]. *forked* (f) regulates the differentiation of epidermal cells and cuticle formation [44]. The kinase *misshapen* (*msn*) regulates cell migration and the epithelial response to wounding [45], and *Mks1* is also involved in epithelial repair [46].

*Schnurri* (*shn*) and Star (*S*) also have roles in regulating tissue differentiation, and both antagonize Notch signaling [47]–[49]. Notch signaling is highly conserved and plays critical roles in cell fate specification. In *Drosophila* it is key to differentiation of crystal cells as vehicles for the prophenoloxidase-activating cascade [50], previously implicated as one of *Drosophila*’s most effective defenses against Ma549 [8].

We found a number of genes involved in hormonal regulation of development, including *taiman* (*tai*), co-activator of the ecdysone regulator [51], *juvenile hormone acid methyltransferase* (*jhmat*), and *Sik3*, a hormone dependent regulator of blood glucose metabolism and starvation responses [52], [53]. Ecdysone mediates the development of immunity in the *Drosophila* embryo [54], and promotes induction of antimicrobial peptides, whereas juvenile hormone is an immuno-suppressor [55]. Juvenile hormone stimulates reproduction at the expense of shorter life span in *Drosophila* [56], opening up the possibility of hormones being regulators of trade-offs between disease resistance and other aspects of fitness at the evolutionary level.

As LT_50_ values correlate with sleep cycles we would expect to find genes that are known to regulate *Drosophila* circadian rhythms. The GABA receptor *Rdl* is a key gene regulating sleep and wake transitions in *Drosophila* [57], [58]. In addition, we found considerable overlap between Ma549 tagged genes and sleep tagged genes from a previous GWAS with the DGRP [9], including, not surprisingly *Rdl* and *hig*, as well as *CG12344*, *CG17209*, *CG32061*, *CG33172*, *CG9990*, *f*, *gem3*, *Nrg*, *S*, *tai* (day average bout length), *Rbp6* (night sleep) and *jhamt*, *msn* (waking activity). *Sickie* and *Rbp6* were also tagged in a screen for parquet-induced oxidative stress [25].

Lastly, several genes had inferred activities (i.e., no experimental evidence) in FlyBase including *Rab26*, a GTPase of no reported function in *Drosophila*, but its mammalian homolog regulates secretion by highly active secretory cells [59]. *Rab26* harbored an SNP with a nominal P-value of P = 1.66 x 10^−7^. Other inferred activities included G-coupled protein receptors (*CG13229*, *CG44153*), a regulator of cell proliferation (*CG33172*), an RNA-binding protein (*Rbp6*), an extracellular-glycine-gated chloride channel (*CG12344*), and an ABC transporter (*CG9990*). Several genes had no known function: *CG13313*, *CG33136*, *CG5111*, *CG8508*, and non-protein coding gene *CR43259*.

To identify genes that confer micro-environmental plasticity to susceptibility to Ma549 infection, we also performed GWA analyses to associate *CV*_*E*_ values with allelic variation. We found 39 SNPs and 7 indels (26 candidate genes) that were associated with micro-environmental plasticity in response to Ma549 at P<10^−5^. A total of five SNPs remained significant when the significance threshold was P<10^−6^. A single gene, *f*, was identified in both this analyses and the GWAS using LT_50_ values.

The screen tagged several cell adhesion molecules involved in the axon guidance system and/or phagocytosis including *Con* [60], *DSCAM* and *DSCAM4* [61], *gukh* [62], and *hdc* [63]. Interestingly, *DSCAM* and *Mhcl* have been studied for their ability to express multiple isoforms suggesting molecular complexity of the systems they influence. Consistent with complexity, *wmd* (muscle morphology), and *Mhcl1* (myosin) are associated with multiple developmental defects in *Drosophila* and have human homologs linked with multisystem diseases [64]–[66]. Likewise, *bab1*, *Xpd* and *qless* have human homologs linked to multiple syndromes [67],[68],[69],[70].

Another notable feature was the number of tagged genes previously implicated in regulating circadian rhythms (*bab1*, *CCKLR-17D3*, *CG10953*, *CG9705*, *Con*, *Ddr*, *DSCAM*, *f*, *gukh*, *CG33687*, and *CG8664*) [71–73]. Most of these genes as well as *CG13917*, *CG13983*, *hdc*, and *Mhcl* were tagged in a screen of genes affecting *CV*_*E*_ of DGRP lines to sleep [9]. As with the LT50 screen, some tagged genes had inferred activities (i.e., no experimental evidence) in FlyBase including *CG14204* (acetyltransferase) and *CG4901* (helicase), and other genes had no known function *CG10953*, *CG13917*, *CG33687*.

### *Pseudomonas aeruginosa* GWAS

Because we terminated the Pa14 bioassay at 14 days, when some of the most resistant fly lines had residual survivors, only the Pa14 mean LT50s were plugged into the DGRP pipeline. Of 62 polymorphisms (P<10^−5^) (51 total associated genes), 12 were indels (7 introns, 4 intergenic and one codon deletion) and 50 were unique SNPs (2 non-synonymous, 7 synonymous, one within 1000 bps downstream, 10 intergenic, two in a 5’UTR, 1 in an exon, and the remaining 27 were intronic). CG42343, a protein coding immunoglobulin-like gene with no known function had 9 significant intronic polymorphisms (4 SNPs, 5 deletions) affecting resistance to Pa14, and all had a negative effect. Each polymorphism that associates significantly with variation in resistance to PA14 is given in S4 Table, including significance level, estimated effect size, minor allele frequency and type of polymorphism.

Surprisingly, given the correlation between Ma549 and Pa14 virulence to DGRP lines, only one tagged gene, *CG44153*, was in common. A similar lack of overlap has been reported in other DGRP studies and this is often attributed to epistasis [14]. Notwithstanding this, Pa14 and Ma549 responsive genes effected many of the same pathways and functions, including Notch signaling and secretion e.g., *CrebA* activates expression of every secretory pathway component gene [74]. Several genes are involved in developmental processes, morphogenesis and tissue maintenance including *Osi1* [75], *Zasp52* [76], G-protein coupled receptor *Mthl1* [77], and *Usp10* (*CG32479*); an ubiquitin specific protease that functions as a positive regulator of notch signaling [78]. Several genes may relate to Pa14’s mode of *per os* infection, including *Mnt* involved in gut cell differentiation and body size [79], and *cert*, which is required for a normal oxidative stress response in the gut [80].

As with Ma549 tagged genes, many of the Pa14 candidates were highly pleiotropic. *Pura* (*CG33275*) is a positive regulator of Rho protein signaling involved in circadian rhythms, perception of pain, and regulation of locomotion [81]. *Ca-alpha1T* is also involved in neural pathways and behavior [82]. *Pde9* has no reported function in *Drosophila*, but its human homolog (63% sequence similarity) is involved in cGMP signaling, hyperglycemia, diabetes, learning, differentiation of stem cells, and neurodegenerative disease [83].

### Gene enrichment and network analysis

We used the DAVID algorithm [84], [85] to perform GO enrichment analysis to assess to what extent the entire suite of candidate genes associated with variation in response to Ma549 and PA14 were functionally related. Using a Benjamini-corrected P < 0.05; GO categories that were significantly enriched for Ma549 resistance (S6 Table) included biological process terms for metamorphosis, morphogenesis, and neuron differentiation indicating that early developmental processes effect subsequent responses to disease. Resistance to PA14 was not associated with significant GO enrichment. We also ran an exploratory GO analysis on genes tagged by polymorphisms using a relaxed p-value of 1x10^-4^ as described [16], [86]. The top Ma549 categories were analogous to GO categories identified at 1x10^-5^ including developmental and morphogenesis genes, but also included cell motion, chemotaxis, cell recognition and cell adhesion, and signaling (S6 Table). Protein domain analysis for either the stringent or the relaxed GO categories displayed an over-representation of immunoglobulin-like genes, fibronectins, and epidermal growth factor-like domains. GO analysis on Pa14 genes tagged by polymorphisms with a p-value of 1x10^-4^ also included morphogenesis, development, adhesion and signaling, with an over representation of immunoglobulin-like genes and fibronectins. These domain analyses suggest that candidate gene with polymorphisms associated with disease resistance include an over representation of extracellular matrix proteins associated with cell adhesion and immunity.

**Fig 10:**
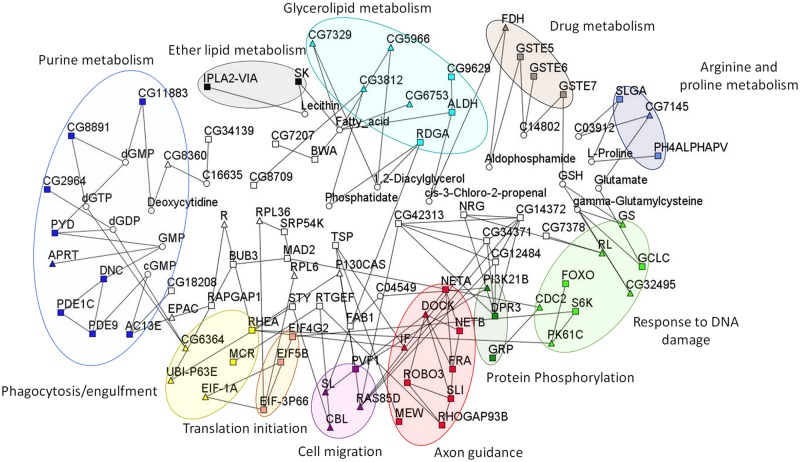
Cellular networks of candidate genes. Enriched cellular genetic pathway for candidate genes from all genome wide association analyses (squares), allowing one missing gene (white triangles) or compound (white circles). The border colors indicate the over-represented gene ontology categories (*P*<0.005): axon guidance (red), translation initiation (orange), protein phosphorylation (dark green), cell migration (magenta), phagocytosis and engulfment (yellow), arginine and proline metabolism (dark blue), purine metabolism (light blue), response to DNA damage (light green), ether lipid metabolism (gray), glycerolipid metabolism (teal), P450 related drug metabolism (brown).

We also used the relaxed p-value of 1x10^-4^ to examine whether these polymorphisms where enriched for true positive associations and cellular networks. To accomplish this, we used the R spider program [87], which organizes gene products into cellular pathways based on the Reactome signaling network and the KEGG metabolic network to determine if interactions are over-represented more than expected by chance. Using Ma549 GWA alone did not produce a significantly enriched network. We therefore performed a network enrichment analysis by pooling all GWA candidate genes associated with resistance to Ma549 (including those that confer micro-environmental plasticity) and Pa14. Using a model that allows for no more than one missing gene or compound, we found a network (P<0.005), comprising 55 candidate genes associated with variation in resistance to Pa14 and Ma549 (Fig 10). The network revealed that genes that harbor alternative natural variation (alleles) associated with susceptibility/resistance are functionally connected through processes that encompass phagocytosis and engulfment, cell mobility, intermediary metabolism (arginine and proline, purine, ether lipids and glycerolipid), protein phosphorylation, axon guidance, response to DNA damage, and cyp450 drug metabolism, which may play a role in detoxification. Many of these genes are well connected in the network, but not all potential connections are included. Thus, transcription factor *FOXO* (4 SNPs tagged in the Ma549 *CV*_*E*_ GWA screen (6.98 x 10^−5^, 2.46 x 10^−5^, 5.67 x 10^−5^, 7.31 x 10^−5^), included in the “Response to DNA damage” domain (Fig 10), is also involved in cross regulation of metabolism and innate immunity [88], and transcriptional regulation for nutrient-stressed flies during resource allocation [89]. Functional validation of the FOXO mutant, showed significantly decreased resistance of the mutant when compared to isogenic control flies (S7 Table). The implication of axon guidance shows that individual variation in susceptibility to pathogens may at least in part be determined by polymorphisms that affect subtle variation in neural function.

### Functional validation of candidate genes

Ten of the random insertional mutations screened previously [8] were in genes tagged in the current Ma549 GWAS screen. Of these six (*Lar*, *msn*, *CG14304*, *CG44153*, *CG14995*, *Rbp6*) had insertional mutations with significant effects on disease resistance, a greater proportion than the 9% expected from random insertional mutations [8].

We additionally used the publicly available toolkit of P-element mutants to confirm the influence of a subset of 13 candidate genes [*S* (Notch signaling), *msn* (response to wounding), *shn* and *CG33172* (cell proliferation), *tai* (ecdysone regulation), *Sik3* (response to starvation), *Rdl* (circadian rhythms), *f* (cuticle formation), *CG9990* (ABC transporter), *CG32066* (unknown function), *CG33111* (unknown function), *puf* (negative regulation of innate immune responses), and *FOXO* (cross regulation of metabolism and innate immunity). These genes were chosen based on the significance level of their association with Ma549, or in the case of FOXO, its detection in a network enrichment analysis. To exclude mutations with generally deleterious effects on fitness we excluded from study genes in which lethal mutations are known. All 13 genes and corresponding controls were tested for both sexes with Ma549.

Nine mutants had significant effects on resistance to Ma549 in both sexes, two mutants (*Sik3* and *CG32066*) had a female-specific effect, one mutant (*puf*) had a male-specific effect, and a mutation in *shn* had no significant effects (S7 Table). In total, 21 out of 26 tests were significant, a much greater proportion than the 9% [8] expected from random insertional mutations (Fisher’s exact test; P = 0.0001), supporting the contention that the top polymorphisms were enriched for true positive associations.

## Discussion

We have utilized two complementary strategies for studies on fungal disease resistance in the fruit fly model system: a mutant screen approach aimed at the characterization of individual candidate genes [8], and in this paper a systems genetic approach to identify natural variation associated with disease resistance. The DGRP has lines that harbor most common variants and a representative sample of rare variants that have survived natural selection, and are unlikely to be produced by mutagenesis screens [9]. Our current study aimed at both quantifying levels of host genetic variation for resistance against different diseases and identifying the specific physiological and genetic factors that influence these traits.

If resistance is defined as an individual’s ability to limit infection by reducing pathogen replication, then disease tolerance can be defined as the ability to limit the impact of infection on a host [90]. To elucidate the underlying mechanisms of variation in LT50 values we determined the fungal loads in hemolymph after infection. Although CFU counts from Ma549-infected insects are clearly affected by the genetic background, flies that succumb to Ma549 only carry high fungal loads in the 36 hours preceding death, irrespective of when this is, suggesting that flies in the more susceptible lines are less able to restrain *Metarhizium* growth. RAL_439 was exceptional in that there was very little fungal proliferation preceding death, suggesting that this line has a physiology that makes it less able to tolerate fungal colonization. Many *Drosophila* mutants succumb to bacterial infections because of defects in tolerance rather than resistance [91], whereas RAL_439 is evidence for genetic variation for tolerance in natural lines. Previously, we suggested based on work with *Drosophila* mutants, that it may be more difficult to evolve tolerance traits to a filamentous fungal pathogen because unlike bacteria they actively penetrate and colonize infected tissues [8]. The present study suggests that most *Drosophila* lines have high tolerance to Ma549, but this is only put to the test in advanced infections when resistance breaks down. The implications of selection for resistance acting in concert with tolerance will need to be considered. Presumably, resistance could lead to selection pressure for higher virulence in the pathogen, whereas selection for tolerance could plausibly result in co-existence of pathogen and host [92].

Susceptibility to Ma549 within the DGRP is sexually dimorphic (Table 1; Fig 1), with males demonstrating higher resistance than females for most lines consistent with our previous mutant screen [8]. This finding is contrary to what is known from most pathogenicity studies in mammals, where females are the more resistant sex. However, exceptions include female mice being more susceptible to *P*. *aeruginosa* infection, showing higher bacterial loads in the lungs [93], so our finding may be part of a broader biological phenomenon. In addition to sexual dimorphism in susceptibility to Ma549 averaged over all DGRP lines, there is also genetic variation in the magnitude and direction of the difference in disease resistance, with females being more resistant than males in about half of the 94 lines that overall are most susceptible to Ma549. More work will be needed in order to understand the molecular mechanisms of these predispositions but they are presumably attributable to multifactorial sex-specific differences in genetics, immune processes, behavior and physiology. We previously demonstrated that virgin and mated females of laboratory *Drosophila* lines have similar susceptibility to Ma549 [8], but that would not necessarily carry over to wild strains.

By studying micro-environmental plasticity, we determined that even a single genotype allows for the production of flies with different susceptibilities to disease, and that this plasticity itself varies depending on genotype. That plasticity is to a large extent under genetic control is shown by its broad sense heritability being twice as high as that of the trait mean for Ma549-induced lethality using the same data set. A similar discordance in heritability values between trait plasticity and mean was obtained for chill-coma recovery time in *Drosophila* [31]. Genetic variation for plasticity will provide the genetic basis of evolution of phenotypic plasticity, making plasticity a heritable trait in its own right and subject to evolutionary mechanisms. Phenotypic plasticity is beneficial in predictably changing environments. The DGRP lines are all derived from an out-crossed population in Raleigh, North Carolina [10], but *Drosophila*’s relatively high migration rate [94], means they may not all have come from the same habitat and be locally adapted to the same pathogens. In these circumstances, phenotypic plasticity to variable pathogen exposures could allow a population to shift from one environment to another without genetic changes, buffering the strength of selection and preventing loss of genetic variation (a “bet-hedging” strategy) [95]. The trait mean and micro-environmental plasticity were highly correlated. Thus, directional selection for an increase in the mean disease resistance will result in more phenotypic variation. If applicable to breeding programs for disease resistance in domestic animals [96] these programs will result in high environmental variance rather than the desired uniformity.

Our results suggest that few of the polymorphisms that contribute to natural variation in resistance to bacteria and fungi in *Drosophila* populations affect canonical immune genes, but rather they cause variation in genes affecting many different aspects of host physiology. These observations are in line with insertional mutagenesis techniques to document resistance genes [8]. Lu [8] reported that 87% of mutated genes in more susceptible lines are involved in a broad spectrum of biological functions not connected with canonical immune systems including basic cellular processes, early development, muscle and nervous system development and function, the senses, and metabolism. Those results are broadly recapitulated in the current GWAS analysis, although there was little overlap in the individual genes implicated by random mutagenesis and natural polymorphisms. The large number of candidate genes involved in development and function of the nervous system is potentially an artifact of the observation that neurological genes tend to be large and therefore provide a larger target for association studies [10], but neurological terms were enriched in our GO analysis that controlled for gene size.

The absence of many canonical immunity genes or immunity related gene ontology terms is of interest as it indicates that these have not been targets of pathogen-dependent selection in the DGRP. Of particular interest, neither Lu [8], nor this study implicate the antifungal peptide genes, although Lu [8] report that drosomyocin is induced by Ma549. One potential explanation is that there is little natural variation in canonical immune genes. However, the DGRP contains 838 variants in the *Toll* gene, 4 in the *Drosomyocin* gene, and 6 in the *Metchnikowin* gene. In contrast, Lu [8] found several indications for octopamine having an immune effect, but that was not replicated in our current GWAS analysis. Octopamine is the key hormone involved in the acute stress response and prepares the insect for flight or fight behaviors, as well as mediating a connection between the nervous system and the immune system [97]. There are several possible and nonmutally exclusive explanations for these observations [98]. In the context of our current study: 1) Naturally occurring polymorphisms may not result in individual variation in resistance responses. This could occur if these genes are under purifying selection and hence functionally invariant. 2) Our GWAS necessarily did not take into account natural selection directed by other pathogens, and specialist pathogens evolving under pairwise co-evolution with *Drosophila* may be more likely to produce signatures of positive selection in immune genes. 3) Effect sizes of causal polymorphisms at these loci are too small to be detected given the resolution of the infection assay and the sample size. 4) Rare alleles at these loci, not interrogated in our analyses, affect natural variation in responses. 5) Functional redundancy in disease resistance genes may obscure the effects of individual polymorphisms on phenotypic variation.

Overall, our results suggest that natural lines differ in their ability to control replicating fungi during infection through the coordinated interplay of morphological restraints and different physiological and immune system effectors. Changes in physiological state likely alter immune-system function via neural/neuroendocrine/immune connections that adapt the immune system to changing needs [6], in subtly different ways in different lines. These results are consistent with studies on domestic animals where the proportion of the total variation in survival explicable by immune variables is too low to be useful as a selection criterion [96]. This was explained by the complexity of the mechanisms involved in the immune response and the large number of factors that may be involved in disease resistance [96]. A majority of polymorphisms associated with disease resistance were intronic, suggesting that gene expression variation may play a major role in determining variability in disease phenotypes. Most were rare suggesting that mutations that increase resistance to Ma549 and PA14 may tend to be deleterious, so are either removed from the population or kept at a low frequency by purifying selection. Our results contrast with a GWAS study which showed that a small number of common polymorphisms have a major effect on resistance to viruses [17]. This may be because there are relatively few genetic changes that can cause viral resistance [17]. Major effect polymorphisms that protect hosts against infection have also been identified in humans [99]–[101], although the majority of human GWAS studies on non-communicable diseases have identified many rare alleles often with small effects [102]. Our association study, like a similar study on sleep in *Drosophila* [9], found that the lower frequency variants had the largest effects (Fig 8), supporting the rare variants hypothesis.

Previous studies have suggested that there is no clear-cut relationship between genetic resistance of *Drosophila* to different bacteria, so a given host genotype does not have a universal effect on a range of bacterial pathogens [103]. Sleiman [28] in their GWAS study found little correlation between enteric infection with *P*. *entomophila* and stab inoculated *Erwinia carotovora*, and concluded that the determinants of gut immunocompetence are distinct from those that govern systemic immunity. Martins [104] also conclude that *Drosophila* adaptation is contingent upon the infection route taken by the pathogen [104]. It was not axiomatic therefore that resistance to a clinical isolate of *P*. *aeruginosa* and Ma549 would be correlated, particularly as bacteria and fungi evoke the IMD and Toll pathways, respectively. Furthermore, we previously found an apparent trade-off in genes affecting resistance to bacterial and fungal infection [8], that was not supported by this finding. The correlation of resistance to PA14 and Ma549 is suggestive of general (multipurpose) defense mechanisms. *Metarhizium* species are abundant in the same soil and plant locations as *Pseudomonas* spp, [105], so local adaptation to these environments will be associated with heightened risk of contracting pathogens.

Depending on the way a pathogen interacts with its host, it may encounter specific or less specific defense barriers. Some of these may also be encountered by other pathogens depending on their routes of entry, host tissues infected and other factors. Fungi infect via the cuticle and bacteria through the gut so these components of the defense machinery will be specific to these pathogens, the unspecific generalized defense components are presumably in host tissues and hemolymph. The host responses triggered by *P*. *aeruginosa* remain poorly understood [106]. However, like *Metarhizium* [107], *P*. *aeruginosa* has means of limiting or resisting antimicrobial peptide gene expression [108]. Our current study shows that resistance to both Ma549 and PA14 correlates with survival times on paraquat but not menadione sodium bisulfite (MSB). Toxicity of paraquat is primarily due to production of superoxides whereas MSB toxicity is due to electrophilic attack [109], suggesting that the ability to alleviate or tolerate superoxide stress is a feature of a generalized defense response to multiple pathogens. *Drosophila* shows circadian regulation of response to oxidative stress [110], so between-line variation in these rhythms may influence how they respond to ROS produced during infection. Circadian control of the immune system is well established in humans [111], and circadian mutants in mice and flies have immune phenotypes [112]. Our data suggests that naturally occurring variation in sleep patterns also impact bacterial and fungal pathogenesis. Given the conservation of both circadian and innate immune signaling between flies and vertebrates, this could have significant implications for vertebrate immunity. It has recently been established that insects can anticipate infections by up-regulating immune genes when they find themselves in scenarios associated with increased disease risk. Zhong et al., [113] raise the interesting possibility that control of immune genes by circadian clock genes might reflect “anticipation” of predictable fluctuations of disease risk over the course of 24 hours. Thus, if frequent naps were associated with pre-emptive up-regulation of immune genes this might be representative of a general pattern of immune anticipation in insects.

Resistance to multiple pathogens should have a selective advantage unless this general defense is traded off against other (pathogen-independent) fitness components [114]. In the absence of such a trade-off, directional selection should lead to fixation of genotypes showing general resistance [115]. However, the most resistant lines to Ma549 were enriched in minor (rare alleles), suggesting that these alleles have negative correlations with other fitness related traits. A trade-off of the cumulative cost of defense could have been reflected by a negative association with longevity and fecundity, which we did not observe. We found some weak positive correlations with blood sugar levels and resistance to starvation stress, consistent with nutritional status altering the quality of immune defense [16]. However, measurements of energy reserves (glycogen stores, total triglycerides and soluble proteins) showed no correlation with disease resistance. This was surprising since Ma549 would compete with hosts for resources, and it makes intuitive sense that overall genotypes that store more nutrients would have better tolerance to disease. Our previous insertional mutagenesis screen showed that half of the mutant lines with altered disease resistance had significant effects on starvation resistance, but there was no simple association between disease and starvation resistance as networks of pleiotropic genes regulate complex traits [8]. Clearly being more or less tolerant to starvation does not by itself alter resistance to Ma549. However, many of the polymorphisms associated with variation in susceptibility to Ma549 are in genes affecting cellular processes and metabolism, and it is plausible that alterations in these processes could specifically change expenditure of energy on immune responses [116].

We found no negative genetic correlations between resistance to Ma549 and several other physiological variables and metabolic indices. Likewise, there were no correlations with measures of ability to cope with important abiotic stresses such as chill-coma recovery time. Southern *Drosophila* populations tend to have higher starvation resistance whereas northern populations tend to have fast chill coma recovery time [117], but our data suggest that this would not be traded off against resistance to Ma549. We did however find examples of genetic variation in the magnitude and direction of associations, such as DGRP lines RAL_399 and RAL_440 that were both highly resistant to Ma549 but demonstrating low and high life time fecundity, respectively. The absence of overall positive or negative correlations between resistance and metabolic indices does not exclude trade-offs as all these parameters were taken by other researchers from uninfected flies, and are complex traits that may not obey simple, single factor models [118]. The lack of a common pattern of correlations among the most resistant or the most susceptible lines i.e., some resistant lines were also particularly resistant to oxidative stress and some were not, suggests that there are multiple mechanisms by which the complex trait of disease resistance can be altered. Consequentially, a GWAS study will identify common trends in populations and not idiosyncratic differences between lines.

With that proviso, our single polymorphism association analyses using the DGRP provided insight into the genetic architecture of susceptibility/resistance to Ma549 associated with variation in this complex trait, and identified novel candidate genes outside the conventional immune system that may be selected for in determining susceptibility to infection. We performed secondary screens using mutations to confirm the reliability of the GWAs in predicting genes that indeed affect disease resistance toward different pathogens. The high validation rate engenders confidence that functional tests of other candidate genes involved in metabolism, development, oxidative stress and function of the nervous system will identify new components of genetic networks affecting disease resistance. The GWA studies presented here are a hypothesis-generating paradigm that lays the foundation for a detailed dissection of allelic effects of candidate genes in future endeavors.

The ecological features that might function as good predictors of host immune investment in *Drosophila* are unknown, but environmental variables, such us parasite species richness, could be informative. For example, fly populations coming from locations with a rich bacterial community have been found to be less susceptible to the bacteria *Lactococcus lactis* [119]. Tinsley [120] found regional differentiation in *Drosophila* susceptibility to the fungus *Beauveria bassiana*, although Paparazzo [121] suggested these differences in susceptibility could be due to general stress resistance. Clearly, much would be gained by being able to integrate our GWA data with studies of ecological genetics in wild *Drosophila* systems that evaluated the process of adaptation to different environments and pathogens [122]. Our current study assessing patterns of variation in host-pathogen interactions improves understanding of the relationship between genetic variation and phenotypic variation for disease resistance, which is necessary for predicting responses to selection. This could have implications for estimating disease risk in humans as several studies have shown the *Drosophila* DGRP can be used to identify functionally similar homologous human genes [9]. It also has implications for development of wild type and genetically engineered entomopathogenic fungi as biocontrol agents of agricultural pests and mosquito vectors of human disease [123]–[125]. Extensive genetic variation in individual resistance from the same geographical population could set the stage for the evolution of resistance with implications for their sustainability. Future studies should also take account of the time of day when applying pathogens to insects in experimental settings or as biocontrol agents, as circadian rhythms may introduce considerable variability.

## Methods

### Growth conditions

The *Drosophila* Genetics Reference Panel (DGRP) [10],[11], and transposon (P-element and Minos insertion) lines were obtained from Bloomington *Drosophila* Stock Center, IN USA. Candidate genes were tested for resistance to fungal infections using insertional mutant fly lines (Bloomington stock number in parenthesis): *S* (20272), *msn* (22796), *shn* (22518), *CG33172* (15945), *tai* (13204), *Sik3* (20921), *Rdl* (26404), *f* (14224), *CG9990* (24814), *CG32066* (16746), *CG33111* (24046), *puf* (15697), and *FOXO*. We received permission to use the *FOXO* mutant from Linda Partridge (University College of London) and the mutant and its control (wDAH) were generously provided by Michael Marr (Brandeis University) [126]. Flies were reared under standard culture conditions (cornmeal-molasses—yeast-agar-medium with Tegosept and propionic acid, 25°C, 12-hr light-dark cycle).

*M*. *anisopliae* (ARSEF 549) was obtained from the USDA Entomopathogenic Fungus Collection (Ithaca, N.Y.). Ma549 is the active ingredient of Metabiol; a commercial product effective against hemipterans, lepidopterans and dipterans, and is a frequently used as a vehicle for genetic engineering projects [124]. Fungal cultures were moved from -80°C stock tubes 10 days before each bioassay and grown on potato dextrose agar at 27°C. Plasmid construction and transformation for the GFP fluorescent Ma549 strain was described previously [123]. *P*. *aeruginosa* (Pa14) was obtained from Vincent Lee (University of Maryland). Bacterial cultures were moved from -80°C stock tubes and plated on LB plates at 37°C two days before each bioassay. Single colonies were moved the next day for overnight growth into flasks containing 25 ml of brain heart infusion (BHI) broth at 37°C and placed on a shaker at 200 rpm.

### Bioassays

Ma549 was used in an infection bioassay as described previously [8]. Flies were maintained at 27°C, 85% humidity, on food made without Tegosept and propionic acid. We bioassayed 3 tubes of ~20 flies (aged 2–4 days) per DGRP line, per sex with a spore suspension (2.5x10^4^ spores/ml of water) produced from 10 day old Ma549 plates. Replicates were run on different days to randomize environmental variation. Control flies were treated with water alone as a control for the bioassay process. Fly mortality was monitored every 12 hrs. We found that in many vials one or two flies died in experimental and control tubes in the first day but subsequently we found that as before [8], there were no differences in longevity between untreated and water treated flies.

A total of 81 randomly chosen DGRP lines were orally infected with PA14 as described in Lutter [127]. Approximately twenty 2–4 day old flies per line, per sex were put into vials and starved without food or water for 5 hrs. During this time, bacterial cultures were normalized to 3.0 at OD600, and 2 ml aliquots centrifuged at 7000 rpm for 5 minutes. The bacterial pellet was suspended in 175 ul of sterile 5% sucrose and then added to 2.3-cm Whatman filter disks placed inside vials containing 6 ml of 5% sucrose agar. Flies were then transferred into the vials and incubated at 27°C and ~85% humidity. Fly mortality was monitored every 12 hrs for 14 days. As *Drosophila* survive night-time infections with *Pseudomonas* significantly better than day-time ones [128], all infections with Ma549 and *Pseudomonas* Pa14 took place within an hour of 6pm.

Time to die was calculated for each replicate tube and expressed as LT_50_. The standard deviation and coefficient of variation for each line were calculated using Kaplan-Meier standard errors. All calculations were done using SPSSv23.

### Quantitative genetic analyses

To assess the effect of *Wolbachia* infection status on survival time to Ma549 and PA14, we used a factorial, type III mixed model ANOVA. The model used was *Y  =  μ+S+I+S×I+L(I)+S×L(I)+ε*, where I denotes the effect of infection status, S is the fixed effect of sex, L is the random effect of the DGRP line, and *ε* is the error variance.

We partitioned phenotypic variance with the ANOVA model *Y* = μ *+S + L + S × L+* ɛ to partition variance among lines (*L*, random), sex (*S*, fixed), line by sex interaction (*L × S*, random), and within-line variance (ɛ). Broad-sense heritabilities for disease resistance were estimated from the variance components as *H*^*2*^ = (σ_*L*_^2^ + σ_*SL*_^2^)/(σ_*L*_^2^ + σ_*SL*_^2^ + σ_*E*_^2^).

To assess the degree of sensitivity of disease resistance to the environment, we first tested the heterogeneity of within line variance among lines using Levene’s test. We then estimated the error mean square separately for each line and replicate by fitting a linear model which only included the intercept (Y = μ + ɛ, where Y is the phenotypic value of the trait, μ is the overall mean and ɛ is the within-replicate random error). We estimated the micro-environmental standard deviation, *σ*_*E*_ as the square root of the mean square errors. We then assessed the genetic variance of ln(*σ*_*E*_) using a mixed model factorial ANOVA model of form Y = u + *L* + ɛ, where Y is ln(*σ*_*E*_), μ is the overall mean, and L is the random effect of the line. Broad sense heritability’s for micro-environmental heterogeneity was calculated as *H*^*2*^ = (σ_*L*_^2^)/(σ_*L*_^2^ + σ_*E*_^2^). All calculations were done using SAS University edition.

### Fungal growth, latent period, and sporulation capacity

Twenty lines were used to survey the impact of fly genetics on Ma549 life history traits. For epifluorescence imaging, ~40 individuals of each line were infected with Ma549-GFP. Fly images were taken starting 12 hours preceding the estimated LT50 for each line, using a Zeiss Axioimager M1. Intact flies were placed on microscope slides underneath a coverslip and viewed at 100x. To view the hemolymph, flies were squashed with the coverslip.

Ten of the lines were selected for a time course bioassay of fungal growth in the hemolymph, using previously described protocols [8]. At each time point, 10 flies per sex were individually homogenized with 45 μl of 0.1% Tween 80. The homogenate was spread onto Rose Bengal Agar plates supplemented with oxbile, CTAP, oxytetracycline, streptomycin, penicillin, chloramphenicol, and cycloheximide. Colony forming units (CFUs) were counted after 7–10 days’ incubation at 25°C.

For sporulation analysis, ten flies per sex were harvested within 12 hours of death and individually transferred into tubes containing a damp cotton ball. The first appearance of spores (latent period) was recorded, and after 20 days, 500 μl of 0.1% Tween 80 was added to each tube and the tubes were vortexed (1 minute). Spore counts per individual fly were made using a hemocytometer, and results are the average of 10 flies per line.

### Phenotypic correlations with other traits

We examined correlations among our measured traits, and between our disease-related phenotypes and independent traits that have been measured in the DGRP lines by other research groups. Correlation analyses were performed in R (R Core Team 2012) using rcorr and our line mean estimates, and we report both correlation coefficient and P value. We used the Holm-Bonferroni method for significant correlations to control for the familywise error rate [129]. For significantly correlated traits, we queried whether a single gene or a few genes might drive the correlation by determining whether the same SNPs were significantly associated with variation in both traits with a P value threshold of 10^−5^.

### GWA analyses

Associations were computed for Ma549 and PA14 separately using line LT50’s and coefficient of variation for phenotypic scores, using ~2 million polymorphic markers [10]. These GWA analyses adjust for the effects of *Wolbachia* infection and 5 major chromosomal inversions (*In(2L)t*, *In(2R)NS*, *In(3R)P*, *In(3R)K*, *In(3R)Mo*), and were implemented using the DGRP website (dgrp2.gnets.ncsu.edu/). The same analysis was performed for each sex separately and for sex average and sex difference of the adjusted phenotypes.

### GO and bioinformatics analysis

Annotation of SNPs was based on Flybase release 5.49 [10]. SNPs were considered in a gene if they were located in or within 1 kb upstream and downstream of a gene model. GO analyses were performed using the DAVID algorithm [84], [85], with the Benjamini correction for multiple tests. To identify ensembles of interacting gene products, we used the R-spider program in the BioProfiling.de web portal [87]). This analysis tool incorporates data for ∼2,000 genes and combines signaling and metabolic pathways from Reactome and Kyoto Encyclopedia of Genes and genomes (KEGG) databases to determine whether interactions between the input genes are greater than expected by chance using a permutation test. The network is built by connecting genes with known interactions in the two databases, allowing zero, one, or two missing nodes.

### Functional tests

P-element insertions in 13 candidate genes were selected for functional assessment, using the criteria that the corresponding polymorphisms had high statistical significance in the GWA analyses, and the mutant alleles were available from *Drosophila* stock collections with co-isogenic controls. We tested *P*-element insertions for their effects on resistance to Ma549 with three to five replicates of approximately 20 flies per line and sex. The *puf* mutant (15697) was originally created using the p-element construct P{EPgy2} which contains a Scer\UAS binding site, inserted into the gene *ash2* [130]. We therefore crossed this line and its isogenic control with a fly line expressing GAL4 (4414) to validate the effect of the *puf* gene. Statistically significant differences in responses to Ma549 between mutants and their coisogenic controls were determined using the log-rank test.

## Supporting information

S1 Table Mean LT_50_ values for Ma549 and PA14, and *Wolbachia* infection status (WI). (DOCX)

S2 Table ANOVA table for *Wolbachia* and common inversions. (XLSX)

S3 Table Absolute rankings for fly lines for different phenotypic traits. (XLSX)

S4 Table GWA analysis results. (XLSX)

S5 Table Ma549 LT50 top association human orthologs (XLSX)

S6 Table Gene ontologies. (XLSX)

S7 Table Mutant validation data. (XLSX)
