# Supplementary material for: The genetic basis for variation in resistance to infection in the Drosophila melanogaster genetic reference panel
Source: PLoS Pathog. 2017 Mar 3;13(3):e1006260. doi: 10.1371/journal.ppat.1006260 (PMC5352145; doi:10.1371/journal.ppat.1006260)
Supplement: S1 Table — (DOCX) [file ppat.1006260.s001.docx]

**S1 Table.** Mean LT_50_  values for Ma549 and PA14, and *Wolbachia* infection status (WI).

|  |  | **Ma549 LT50** | | **Pa14 LT50** | |
| --- | --- | --- | --- | --- | --- |
| **DGRP** | **WI** | **Males** | **Female** | **Male** | **Female** |
| RAL_21 | y | 4.33 | 4.836 | 3.956 | 3.687 |
| RAL_26 | n | 4.465 | 5.073 | NA | NA |
| RAL_28 | n | 5.248 | 4.966 | NA | NA |
| RAL_31 | n | 6.032 | 5.428 | 5.222 | 6.993 |
| RAL_32 | n | 4.733 | 4.274 | NA | NA |
| RAL_38 | n | 7.048 | 6.478 | 3.904 | 4.591 |
| RAL_40 | y | 5.886 | 5.297 | NA | NA |
| RAL_41 | n | 5.442 | 5.385 | NA | NA |
| RAL_42 | n | 5.069 | 4.493 | NA | NA |
| RAL_45 | n | 6.589 | 5.941 | 3.313 | 4.308 |
| RAL_48 | y | 7.02 | 5.849 | 6.664 | 5.585 |
| RAL_57 | n | 5.931 | 5.581 | NA | NA |
| RAL_59 | n | 5.528 | 5.319 | 4.263 | 4.515 |
| RAL_69 | y | 4.585 | 4.974 | NA | NA |
| RAL_73 | y | 4.055 | 3.872 | 3.481 | 4.618 |
| RAL_75 | y | 5.179 | 4.596 | NA | NA |
| RAL_83 | n | 5.956 | 4.702 | NA | NA |
| RAL_85 | n | 6.31 | 5.203 | 4.313 | 2.998 |
| RAL_88 | n | 5.151 | 4.969 | NA | NA |
| RAL_91 | n | 5.447 | 4.688 | NA | NA |
| RAL_93 | n | 5.897 | 5.735 | NA | NA |
| RAL_100 | y | 5.684 | 4.902 | NA | NA |
| RAL_101 | n | 6.481 | 5.353 | 7.349 | 6.547 |
| RAL_105 | n | 6.699 | 5.697 | NA | NA |
| RAL_129 | n | 4.674 | 4.808 | NA | NA |
| RAL_136 | y | 5.863 | 5.807 | 7.323 | 6.703 |
| RAL_138 | n | 5.744 | 5.08 | NA | NA |
| RAL_142 | y | 5.68 | 5.012 | NA | NA |
| RAL_149 | y | 4.377 | 4.686 | 1.527 | 1.529 |
| RAL_153 | y | 4.94 | 4.818 | NA | NA |
| RAL_158 | n | 5.003 | 4.936 | NA | NA |
| RAL_176 | y | 5.448 | 6.136 | 1.989 | 2.428 |
| RAL_177 | n | 4.871 | 4.673 | NA | NA |
| RAL_181 | y | 4.707 | 4.854 | NA | NA |
| RAL_195 | n | 5.145 | 5.261 | NA | NA |
| RAL_208 | n | 5.995 | 5.29 | NA | NA |
| RAL_217 | n | 5.133 | 4.476 | NA | NA |
| RAL_227 | y | 3.752 | 3.788 | 3.219 | 3.363 |
| RAL_228 | n | 5.86 | 5.235 | 4.764 | 3.982 |
| RAL_229 | n | 5.835 | 4.981 | 5.555 | 4.365 |
| RAL_235 | n | 5.413 | 4.904 | NA | NA |
| RAL_237 | y | 4.945 | 4.582 | NA | NA |
| RAL_239 | n | 4.599 | 4.316 | 1.059 | 1.068 |
| RAL_256 | y | 5.11 | 4.999 | 4.345 | 2.911 |
| RAL_280 | y | 5.24 | 4.518 | NA | NA |
| RAL_287 | y | 5.102 | 5.043 | 1.631 | 1.175 |
| RAL_301 | n | 6.025 | 5.841 | 5.224 | 3.834 |
| RAL_303 | n | 4.877 | 4.811 | NA | NA |
| RAL_304 | y | 5.095 | 5.204 | NA | NA |
| RAL_306 | y | 5.142 | 5.286 | NA | NA |
| RAL_307 | n | 4.702 | 4.409 | 2.552 | 3.093 |
| RAL_309 | n | 5.198 | 4.254 | 1.747 | 1.972 |
| RAL_310 | y | 6.669 | 6.041 | 5.915 | 5.136 |
| RAL_313 | n | 4.508 | 4.789 | NA | NA |
| RAL_315 | n | 6.026 | 5.552 | NA | NA |
| RAL_317 | y | 6.287 | 5.621 | 4.665 | 3.307 |
| RAL_318 | y | 5.018 | 4.509 | NA | NA |
| RAL_319 | y | 5.62 | 5.334 | NA | NA |
| RAL_320 | y | 5.727 | 5.166 | 5.315 | 4.698 |
| RAL_321 | y | 3.888 | 3.549 | 3.873 | 3.62 |
| RAL_324 | n | 5.052 | 4.149 | 6.502 | 4.409 |
| RAL_332 | n | 3.985 | 3.814 | NA | NA |
| RAL_335 | y | 4.052 | 4.266 | 3.137 | 3.469 |
| RAL_336 | y | 5.643 | 4.924 | NA | NA |
| RAL_338 | y | 5.196 | 5.972 | NA | NA |
| RAL_340 | y | 4.85 | 4.739 | NA | NA |
| RAL_348 | n | 5.139 | 4.864 | 3.452 | 2.875 |
| RAL_352 | y | 5.157 | 4.674 | NA | NA |
| RAL_354 | n | 5.557 | 5.274 | NA | NA |
| RAL_356 | y | 5.256 | 5.341 | NA | NA |
| RAL_357 | n | 5.711 | 5.164 | 7.469 | 4.225 |
| RAL_358 | n | 4.506 | 4.758 | NA | NA |
| RAL_359 | n | 4.965 | 4.534 | NA | NA |
| RAL_360 | y | 4.433 | 4.221 | 2.236 | 3.084 |
| RAL_361 | y | 5.127 | 5.544 | NA | NA |
| RAL_362 | y | 6.859 | 5.801 | 5.704 | 4.714 |
| RAL_365 | y | 4.978 | 4.784 | NA | NA |
| RAL_367 | n | 5.059 | 4.523 | 3.059 | 4.901 |
| RAL_370 | y | 5.238 | 4.894 | NA | NA |
| RAL_371 | n | 5.639 | 5.139 | NA | NA |
| RAL_373 | n | 4.992 | 4.744 | NA | NA |
| RAL_375 | n | 4.88 | 5.031 | NA | NA |
| RAL_377 | n | 5.068 | 5.513 | NA | NA |
| RAL_379 | n | 4.893 | 5.47 | NA | NA |
| RAL_380 | y | NA | NA | 4.205 | 2.759 |
| RAL_381 | n | 3.998 | 4.53 | NA | NA |
| RAL_382 | y | 5.125 | 5.243 | NA | NA |
| RAL_383 | y | 5.085 | 4.764 | NA | NA |
| RAL_385 | n | NA | NA | 4.675 | 3.172 |
| RAL_386 | n | 4.627 | 4.225 | 2.797 | 2.725 |
| RAL_390 | n | 4.369 | 4.355 | 5.013 | 3.709 |
| RAL_391 | n | 5.713 | 5.419 | NA | NA |
| RAL_392 | n | 5.072 | 4.723 | NA | NA |
| RAL_395 | n | 6.112 | 5.724 | 3.242 | 3.378 |
| RAL_397 | y | 3.924 | 4.463 | 1.663 | 1.921 |
| RAL_399 | n | 6.858 | 6.29 | 4.459 | 4.485 |
| RAL_405 | y | 6.573 | 6.809 | 5.427 | 3.909 |
| RAL_406 | n | 5.393 | 5.181 | NA | NA |
| RAL_409 | y | 4.494 | 4.2 | 3.197 | 2.621 |
| RAL_426 | n | 4.189 | 4.532 | 5.732 | 5.318 |
| RAL_427 | n | 5.004 | 4.922 | NA | NA |
| RAL_437 | n | 4.469 | 4.539 | NA | NA |
| RAL_439 | n | 3.732 | 4.281 | NA | NA |
| RAL_440 | y | 6.018 | 6.251 | 3.3 | 2.631 |
| RAL_441 | y | 4.824 | 4.731 | NA | NA |
| RAL_443 | n | 6.926 | 5.721 | 6.029 | 6.624 |
| RAL_461 | y | 4.64 | 4.238 | NA | NA |
| RAL_486 | y | 6.085 | 5.72 | NA | NA |
| RAL_491 | n | 4.971 | 5.054 | NA | NA |
| RAL_492 | n | 4.334 | 4.868 | 2.294 | 3.633 |
| RAL_502 | n | 4.877 | 4.098 | 3.029 | 2.815 |
| RAL_508 | n | 4.902 | 4.279 | 4.722 | 4.917 |
| RAL_509 | n | 5.561 | 5.2 | NA | NA |
| RAL_513 | y | 4.941 | 4.8 | 3.091 | 2.671 |
| RAL_517 | n | 5.274 | 4.486 | 7.271 | 5.267 |
| RAL_528 | y | 4.967 | 5.085 | NA | NA |
| RAL_530 | y | 6.002 | 4.765 | NA | NA |
| RAL_535 | y | 5.446 | 5.859 | 3.546 | 3.366 |
| RAL_551 | y | 5.772 | 5.094 | NA | NA |
| RAL_555 | y | 6.5 | 5.368 | 3.371 | 2.305 |
| RAL_559 | n | 4.855 | 4.646 | NA | NA |
| RAL_563 | n | 6.042 | 5.28 | NA | NA |
| RAL_566 | n | 5.471 | 5.443 | NA | NA |
| RAL_584 | y | 4.626 | 5.085 | NA | NA |
| RAL_589 | y | 5.344 | 5.821 | 4.676 | 5.465 |
| RAL_595 | y | 4.762 | 4.227 | 4.202 | 1.522 |
| RAL_627 | n | 4.576 | 4.711 | 3.834 | 2.749 |
| RAL_630 | n | 5.586 | 5.037 | NA | NA |
| RAL_634 | y | 5.602 | 5.016 | NA | NA |
| RAL_639 | y | 4.104 | 5.052 | 3.938 | 3.139 |
| RAL_642 | n | 5.453 | 5.295 | NA | NA |
| RAL_646 | y | 5.568 | 5.129 | NA | NA |
| RAL_703 | n | 5.198 | 4.935 | NA | NA |
| RAL_705 | y | NA | NA | 7.641 | 4.413 |
| RAL_707 | y | 5.65 | 5.043 | NA | NA |
| RAL_712 | y | 4.712 | 5.093 | NA | NA |
| RAL_714 | n | 5.103 | 4.731 | 1.725 | 1.339 |
| RAL_716 | y | 5.679 | 5.175 | NA | NA |
| RAL_721 | y | 4.533 | 4.702 | NA | NA |
| RAL_727 | y | 6.335 | 5.182 | 6.015 | 5.432 |
| RAL_730 | y | 5.741 | 5.837 | NA | NA |
| RAL_732 | n | 6.436 | 5.712 | 2.978 | 4.032 |
| RAL_737 | y | 4.09 | 4.56 | 4.985 | 4.515 |
| RAL_738 | y | 5.42 | 4.939 | NA | NA |
| RAL_748 | y | 6.497 | 6.73 | 3.131 | 4.618 |
| RAL_757 | n | 4.189 | 4.507 | 0.967 | 1.144 |
| RAL_761 | y | 5.342 | 5.139 | NA | NA |
| RAL_765 | n | 5.504 | 4.962 | NA | NA |
| RAL_774 | n | 5.499 | 5.161 | NA | NA |
| RAL_776 | y | 5.823 | 5.316 | NA | NA |
| RAL_783 | y | 4.829 | 4.294 | 4.686 | 2.624 |
| RAL_786 | y | 4.1 | 4.557 | 3.253 | 3.122 |
| RAL_787 | y | 4.196 | 4.211 | 4.655 | 3.789 |
| RAL_790 | y | 4.071 | 4.506 | 3.015 | 3.821 |
| RAL_796 | y | 6.325 | 5.466 | 8.323 | 6.981 |
| RAL_799 | n | 6.557 | 5.024 | 2.449 | 4.268 |
| RAL_801 | y | 5.528 | 5.225 | NA | NA |
| RAL_802 | y | 5.438 | 4.972 | NA | NA |
| RAL_804 | y | 5.97 | 4.971 | NA | NA |
| RAL_805 | y | 4.611 | 4.399 | NA | NA |
| RAL_808 | n | 6.947 | 6.213 | 5.63 | 4.963 |
| RAL_810 | n | 5.544 | 5.47 | NA | NA |
| RAL_812 | n | 5.267 | 5.999 | 5.26 | 4.456 |
| RAL_818 | y | 5.11 | 4.766 | NA | NA |
| RAL_819 | y | 5.633 | 5.526 | NA | NA |
| RAL_820 | y | 5.25 | 5.48 | NA | NA |
| RAL_821 | y | 5.609 | 4.756 | NA | NA |
| RAL_822 | y | 4.976 | 6.11 | 3.393 | 4.637 |
| RAL_832 | y | 4.186 | 4.723 | 2.314 | 1.794 |
| RAL_837 | y | 5.593 | 4.908 | NA | NA |
| RAL_843 | n | 5.358 | 4.915 | NA | NA |
| RAL_849 | n | 4.83 | 5.12 | NA | NA |
| RAL_850 | y | 4.887 | 4.957 | NA | NA |
| RAL_852 | y | 5.187 | 5.624 | 2.389 | 4.557 |
| RAL_853 | y | 5.636 | 5.819 | 6.505 | 5.361 |
| RAL_855 | y | 6.16 | 5.635 | 4.731 | 5.205 |
| RAL_859 | y | 5.635 | 4.922 | NA | NA |
| RAL_861 | y | 6.077 | 5.187 | NA | NA |
| RAL_879 | y | 4.788 | 4.734 | 4.036 | 3.363 |
| RAL_882 | y | 6.554 | 5.555 | 5.311 | 6.556 |
| RAL_884 | y | 5.948 | 5.617 | NA | NA |
| RAL_887 | y | 5.641 | 4.931 | NA | NA |
| RAL_890 | y | 6.622 | 5.673 | 5.213 | 3.874 |
| RAL_892 | y | 5.174 | 4.593 | 1.867 | 1.188 |
| RAL_894 | n | 5.699 | 4.666 | NA | NA |
| RAL_897 | y | 4.556 | 4.845 | NA | NA |
| RAL_900 | n | 4.831 | 5.298 | NA | NA |
| RAL_907 | n | 4.882 | 5.148 | NA | NA |
| RAL_908 | n | 5.434 | 5.484 | NA | NA |
| RAL_911 | n | 6.218 | 5.884 | 3.035 | 2.633 |
| RAL_913 | y | 6.291 | 5.806 | 9.199 | 6.853 |
